# Natural Coinfection with 2 Parvovirus Variants in Dog

**DOI:** 10.3201/eid1404.071461

**Published:** 2008-04

**Authors:** Maria João Vieira, Eliane Silva, Costantina Desario, Nicola Decaro, Júlio Carvalheira, Canio Buonavoglia, Gertrude Thompson

**Affiliations:** * Universidade do Porto, Porto, Portugal; † Clinicão-Clínica Veterinária, Figueira da Foz, Portugal; ‡ University of Bari, Bari, Italy

**Keywords:** Canine parvovirus, coinfection, virus variants, PCR-RFLP, MGB probe assays, gastroenteritis, letter

**To the Editor:** Canine parvovirus (CPV) emerged in the late 1970s, presumably by mutations in feline panleukopenia virus, and became a major viral pathogen of dogs worldwide (*1*). Between 1979 and 1981, the original type 2 virus (CPV-2) was replaced by a new genetic and antigenic variant, type 2a (CPV-2a). Between 1983 and 1984, CPV-2a was replaced by type 2b (CPV-2b), which differs from type 2a by only 1 epitope located at residue 426 of the VP2 capsid protein (*2*). CPV-2 does not replicate in cats, but the new variants replicate in dogs and cats (*3*). Recently, an antigenic change has been observed in a new strain, CPV-2c, isolated from domestic dogs in Italy (*4*). This variant was also detected in Vietnam (*5*), other countries in Europe (*6*), and the United States (*7*). CPV-2c was recently detected in cats (*8*) and is characterized by a replacement of aspartic acid with glutamic acid at residue 426 of the VP2 capsid protein.

To identify CPV types 2, 2a, and 2b, PCR methods were developed (*9*). However, these methods could not distinguish type 2c from type 2b (*4*). Consequently, we used a PCR–restriction fragment-length polymorphism (RFLP) assay with endonuclease *Mbo*II. This enzyme can distinguish type 2c from other CPVs (*4*). Recently, a real-time PCR assay based on minor groove binder (MGB) probe technology was developed for rapid identification and characterization of the antigenic variants. This assay is based on 1 nucleotide polymorphism in the VP2 gene (*10*).

In June 2006, a 10-week-old female dog (PT-32/07) was brought to the veterinary clinic in Figueira da Foz, Portugal, with clinical signs of parvovirus infection, after an episode of gastrointestinal disease in her littermates. Three littermates, also brought to the clinic, showed no signs of infection. None of the dogs were vaccinated against CPV. Clinical signs in dog PT-32/07 were lethargy, anorexia, vomiting, diarrhea, and a temperature of 39.3°C. Identical signs were observed in 1 littermate 3 days later; the 2 other dogs did not show any signs other than lethargy and loose stools.

Rectal swab samples from all dogs were screened for CPV by using an immunomigration rapid test (Synbiotics Corporation, Lyon, France). Two of the dogs showed negative results, and 2 showed positive results. Feces, serum, and lingual swab samples were positive for parvovirus DNA. DNA was quantified by using a real-time PCR with TaqMan technology performed in an i-Cycler iQ (Bio-Rad Laboratories, Milan, Italy).

CPV variants were characterized by using MGB probe technology. This technology uses type-specific probes labeled with different fluorophores (FAM and VIC) that can detect single nucleotide polymorphisms between CPV types 2a/2b and 2b/2c (*10*). MGB probes specific for type 2b were labeled with FAM in both type 2a/2b and 2b/2c assays, and MGB probes specific for type 2a (type 2a/2b assay) and type 2c (type 2b/2c assay) were labeled with VIC.

All specimens from 1 dog (PT-32/07) were positive for the 2 variants of CPV type 2 (CPV 2b and CPV 2c). Conversely, of the 3 littermates, 2 were positive for CPV type 2b and 1 was positive for CPV type 2c in all samples (Table).

**Table:** Detection by minor groove binder probe assay of CPV antigenic variants in different specimens from dogs from the same litter (10 weeks old), Portugal, 2006*

Dog	Vaccines	Rapid test result for CPV	Days in clinic	Clinical course	Samples	TaqMan probe				CPV
						FAM a/b	FAM b/c	VIC a/b	VIC b/c	
PT-15/07	None	–	7	Recovered	Feces	+	+	–	–	2b
					Lingual swab	+	+	–	–	2b
					Serum	+	+	–	–	2b
PT-16/07	None	+	7	Recovered	Feces	+	+	–	–	2b
					Lingual swab	+	+	–	–	2b
					Serum	+	+	–	–	2b
PT-17/07	None	+	7	Recovered	Feces	–	–	–	+	2c
					Lingual swab	–	–	–	+	2c
					Serum	–	–	–	+	2c
PT-32/07	None	–	7	Recovered	Feces	+	+	–	+	2b/2c
					Lingual swab	+	+	–	+	2b/2c
					Serum	+	+	–	+	2b/2c

*CPV, canine parvovirus.

A conventional PCR and RFLP analyses were performed by using the method of Buonavoglia et al. (*4*) with known positive CPV-2b and CPV-2c samples as controls to confirm our findings. The 583-bp PCR product obtained from the coinfected dog by using primer pair 555for/555rev was digested with *Mbo*II. Digestion generated 2 fragments (≈500 and 80 bp) in all dog samples. The CPV-2c control sample showed 2 fragments (≈500 and 80 bp), and CPV-2b control sample was not digested with *Mbo*II.

We report CPV-2b and CPV-2c variants in samples from a dog with littermates that were positive for CPV-2b or CPV-2c during an episode of gastrointestinal disease. Coinfection with multiple CPV variants that showed high genetic diversity in the VP2 gene has recently been reported in a domestic cat (*8*). Continuous and rapid evolution of CPV may cause serious problems in diagnostic testing and vaccine efficacy. Antigenic variation may negatively affect vaccine efficacy if changes occur at major antigenic sites. Thus, continuous monitoring for novel genetic and antigenic virus types is needed. Additional studies are in progress to characterize nucleotide sequences of all CPV isolates from this case.
